# The Multi Domain *Caldicellulosiruptor bescii* CelA Cellulase Excels at the Hydrolysis of Crystalline Cellulose

**DOI:** 10.1038/s41598-017-08985-w

**Published:** 2017-08-29

**Authors:** Roman Brunecky, Bryon S. Donohoe, John M. Yarbrough, Ashutosh Mittal, Brian R. Scott, Hanshu Ding, Larry E. Taylor II, Jordan F. Russell, Daehwan Chung, Janet Westpheling, Sarah A. Teter, Michael E. Himmel, Yannick J. Bomble

**Affiliations:** 1 Biosciences Center, National Renewable Energy Laboratory, 15013 Denver West Parkway, Golden, CO 80401 USA; 2 Novozymes, Inc., 1445 Drew Ave, Davis, CA 95618 USA; 3 Department of Genetics, University of Georgia, Athens, GA 30602 USA

## Abstract

The crystalline nature of cellulose microfibrils is one of the key factors influencing biomass recalcitrance which is a key technical and economic barrier to overcome to make cellulosic biofuels a commercial reality. To date, all known fungal enzymes tested have great difficulty degrading highly crystalline cellulosic substrates. We have demonstrated that the CelA cellulase from *Caldicellulosiruptor bescii* degrades highly crystalline cellulose as well as low crystallinity substrates making it the only known cellulase to function well on highly crystalline cellulose. Unlike the secretomes of cellulolytic fungi, which typically comprise multiple, single catalytic domain enzymes for biomass degradation, some bacterial systems employ an alternative strategy that utilizes multi-catalytic domain cellulases. Additionally, CelA is extremely thermostable and highly active at elevated temperatures, unlike commercial fungal cellulases. Furthermore we have determined that the factors negatively affecting digestion of lignocellulosic materials by *C. bescii* enzyme cocktails containing CelA appear to be significantly different from the performance barriers affecting fungal cellulases. Here, we explore the activity and degradation mechanism of CelA on a variety of pretreated substrates to better understand how the different bulk components of biomass, such as xylan and lignin, impact its performance.

## Introduction

*Caldicellulosiruptor bescii*, a hyperthermophilic anaerobic bacterium, is one of the most cellulolytic bacteria in the biosphere^1^. It was first isolated from the Valley of Geysers region in Siberia and quickly showed promise as an efficient biomass degrader^1^. C. *bescii*, unlike most other cellulolytic bacteria, utilizes primarily multi-modular, multi-functional cellulases to degrade plant cell walls. These more complex extracellular enzymes are comprised of at least two catalytic domains. Among these enzymes, CelA is the most prevalent and one of the most important cellulases secreted into the *C. bescii* exoproteome. The deletion of the *celA* gene results in significantly decreased cell growth on biomass; as well as a significant reduction in the cellulolytic activity of the exoproteome acting on crystalline cellulose^2^. CelA is a complex, thermally stable cellulase, containing an *N*-terminal family 9A-CBM3 processive endoglucanase, two family 3 carbohydrate-binding modules (CBM3), and a *C*-terminal GH48 exo-β-1,4-glucanase domain, first isolated by Zverlov *et al*.^1^.

In our initial characterization of CelA, we reported its impressive performance on simple cellulose substrates. Additionally, the efficient degradation of the model cellulose, Avicel, exhibited a novel deconstruction mechanism distinct from other cellulases, wherein CelA uses not only the common surface ablation strategy driven by processivity, but also digs cavities that penetrate well beyond the surface of the Avicel particles^3^. Furthermore, CelA employs two catalytic domains, each acting on different ends of cellulose (e.g., the reducing and non-reducing). This combination of deconstruction mechanisms is especially effective on simple cellulose substrates, but the conversion of more complex and recalcitrant whole biomass varies widely^3^. In this current work, we have continued investigating the mechanisms of CelA and have found several important differences between CelA and canonical fungal cellulase systems including the well studied Cel7a family^4–9^. These differences include differential responses to cellulose properties like crystallinity, and enzyme interactions with cell wall matrix polymers including lignin and xylan which typically have negative effects on the digestion of biomass^10–15^. These additional substrate factors determine the recalcitrance of biomass and can dominate the interaction of cellulolytic enzyme systems with whole biomass. Physical and/or chemical pretreatments improve saccharification of cellulose in lignocellulosic feedstocks by modifying and partially solubilizing hemicellulose and lignin and increasing cellulose accessibility. Dilute acid pretreatment of corn stover was shown to markedly increase cellulose saccharification by fungal cellulases but had little effect on digestion by CelA^3^. Taken together with the novel cavity-forming mechanism demonstrated previously for CelA on Avicel, this implies that at least some of the barriers to efficient saccharification of lignocellulose are different for CelA compared to fungal cellulases. Here, we aim to explain how these factors affect the deconstruction of biomass by CelA.

## Results

### CelA is more effective at deconstruction of biomass pretreated with chemistries that drastically reduce lignin content

To further investigate the effects of substrate composition on CelA activity, we differentially pretreated substrates to obtain different bulk chemical properties generated from the same corn stover lot. The dilute acid (DA) treated corn stover is comprised primarily of glucan and lignin, with a minimal remaining xylan content. The alkaline peroxide (AP) treated corn stover is primarily comprised of glucan and xylan, with little lignin remaining, and the Clean Fractionation (CF) process treated corn stover contains primarily glucan, with a minimal amount of xylan or lignin content (Table S1). These differentially treated corn stover samples were digested with either a hydrophobically enriched *C. bescii* broth (Cb Broth containing mostly the enzyme CelA) or a free enzyme mix (Cellic® CTec2, Novozymes) - comprised of multiple components with a Cel7A being the dominant species. Incubations were done at the temperature optimum of each enzyme system, 75 °C for the Cb broth and 50 °C for CTec2.

A kinetic model was then used to fit the hydrolysis time course data and quantify differences in the kinetics of Cb broth and CTec2 on these pretreated substrates. The model and its application to the data are described in the Materials and Methods. In the first step, active cellulases (E_a_) catalyze the conversion of cellulose (S) to cellobiose (G2) and in the second step β-glucosidase catalyzes the conversion of cellobiose to glucose (G). Cellulases are assumed to be subject to competitive inhibition by glucose, cellobiose and lignin (L) and subject to time-dependent inactivation. β-glucosidase is assumed to be subject to competitive inhibition by glucose. In applying the model to the data, seven of the nine parameters were fixed using the values shown in Table 1. The model was fit to the data by varying the cellulase inactivation rate constant (*k*_i_) and either the cellulase catalytic rate constant (*k*_s_) or the lignin inhibition constant (K_L_). Therefore, differences in the apparent initial rate of cellulose hydrolysis were captured in either *k*_s_ or K_L_ while time-dependent differences in hydrolysis performance were captured in *k*_i_. The choice of this model and potential challenges of the assumptions therein are addressed in the Materials and Methods and in the Discussion.

**Table 1:** Values for individual kinetic parameters that were fixed in all model fits.

Parameter	Value
K_s_	42 g/L
K_G_^a^	13 g/L
K_G2_	3 g/L
*k*_cat_	100 h^−1^
K_M_	2 g/L
K_G_^b^	1 g/L

**Figure 1:**
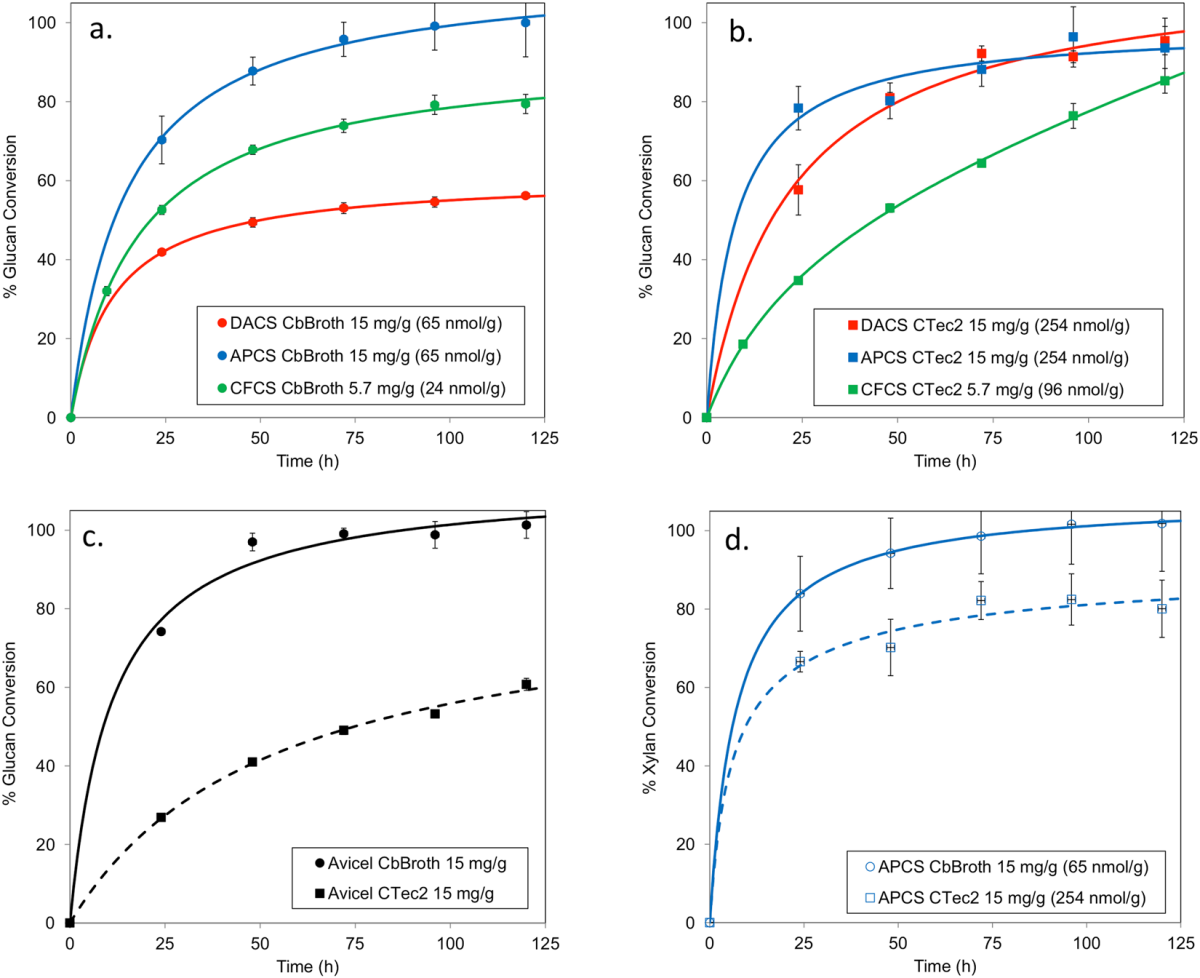
(**a,b**) Activity of Cb broth and CTec2, respectively, on Dilute Acid treated Corn Stover, on alkaline peroxide treated corn stover and on Clean fractionated corn stover (**c**) Conversion of Avicel by Cb broth and CTec2. (**d**) Xylan conversion of Cb broth and CTec2 of alkaline peroxide treated corn stover.

The enzyme activity curves in Fig. 1 show that Cb broth has a clear preference for substrates that contain minimal lignin; performing the best on the CF and AP treated (Fig. 1a,d) materials. The values of catalytic rate constant, *k*_s_, (54, 55 and 47 h^−1^, respectively) were very similar on CFCS, APCS and Avicel, respectively, while the value of *k*_i_ was fixed for these substrates (Table 2). Clearly the substantial level of remaining xylan in APCS and low levels of remaining lignin in both the APCS and CFCS have negligible impact on Cb broth. However, Cb broth was considerably less effective on the DA treated substrate (Fig. 1a), which contains markedly higher levels of lignin. Given the remarkably consistent *k*_s_ observed on the other substrates, when modeling Cb broth data on DACS, the value of *k*_s_ was fixed to 54 h^−1^, the same value estimated on CFCS, and the model fit to the data by varying the lignin inhibition constant, K_L_, and *k*_i_. This analysis demonstrated that inactivation of Cb broth increased approximately 2-fold on DACS compared to CFCS. In addition, the low value of K_L_ (1.1 g/L) implies substantial inhibition of Cb broth enzymes by lignin.

**Table 2:** CelA and CTec2 were tested on DACS, APCS, CFCS, and Avicel and the kinetic parameters from these model fits are shown.

Substrate	Enzyme	*k*_s_ (h^−1^)	K_L_ (g/L)	*k*_i_ (×10^−2^ h^−1^)	t_1/2_ (h)	t_1/2_ 95% CI (h)
DACS	Cb broth	54^a^	1.1	4.4	16	13–18
DACS	CTec2	23 ± 3	100^a^	0.3	206	54-
APCS	Cb broth	55 ± 7	100^a^	2.2^b^	31	—
APCS	CTec2	59 ± 54	100^a^	3.0	23	7–142
CFCS	Cb broth	54 ± 2	100^a^	2.2^b^	31	—
CFCS	CTec2	20 ± 2	100^a^	0	n/a	169-
Avicel	Cb broth	47 ± 4	100^a^	2.2^b^	31	—
Avicel	CTec2	6 ± 1	100^a^	1.1	65	49–92

The model was fit to each progress curve shown in Fig. S3 by varying *k*_i_ and *k*_s_ or K_L_. Values shown are best fit values and either their associated standard deviations or 95% confidence intervals.

^a^Value was fixed while estimating values for remaining parameters.

^b^Value was estimated using a global fit to CelA data on CFCS, APCS and Avicel.

The fungal free enzyme system, CTec2, displayed greater tolerance to the lignin content of the pretreated substrates, as the kinetic parameters estimated for CTec2 were remarkably similar on CFCS and DACS. However, CTec2 showed slightly slower rates of conversion on the CF and AP treated substrates compared to the Cb broth (Fig. 1b,d). Interestingly, the free enzyme system is challenged when converting Avicel to sugars, as the catalytic rate constants for CTec2 on CFCS (20 h^−1^) and APCS (59 h^−1^) are substantially higher than on Avicel (6 h^−1^). Given the relatively low levels of xylan and lignin in the CFCS, it is highly plausible that the increases in catalytic rate observed here for CTec2 are associated with increased accessibility of the fungal cellulases to the cellulose in these substrates. This is a known performance barrier for free enzyme systems that does not appear to affect Cb broth, as the hydrolysis kinetics were similar on both Avicel and CFCS (Figure S3).

In the case of xylan conversion, the Cb broth achieved a superior extent of conversion on the AP treated material (Fig. 1d), but an inferior level of conversion compared to the free enzyme mixture when acting on the DA treated material. This result may reflect a more recalcitrant xylan moiety remaining after dilute acid pretreatments due to the highly substituted nature of xylans in grasses. Moreover, these xylans may also be complexed with a lignin as so-called lignin/carbohydrate complexes (LCCs).

It should be noted that in all of the digestion experiments presented here, the enzyme loading was carried out on an equivalent mass basis (mg enzyme per g/glucan). This method of measure is convenient, consistent, and industrially relevant. However, CelA (the main component of the Cb broth) and other *C. bescii* multi-modular cellulases are much larger than their free-fungal counterparts, having roughly four fold more mass compared to free-fungal enzymes. Consequently, when considering the experiments as presented here, the actual molar loadings of all of the *C. bescii* based mixes are roughly four-fold lower when compared to the free fungal enzyme mixes, and roughly two-fold lower when considered in terms of an equivalent number of active sites present^3^.

### Cellulose crystallinity is not an important performance barrier to the activity of CelA

Cb broth greatly outperforms CTec2 on the model substrate, Avicel, which is known to have higher crystallinity than native cellulose (Fig. 1c). To examine the effect of cellulose crystallinity on the activity of CelA and fungal enzymes, we generated three cellulose samples with varying degrees of crystallinity index (CI) (e.g., 66%, 45% and 33%). We digested these substrates using both a fungal Cel7A + E1 + BG combination (a Cel7A being the dominant enzyme in CTec2 we selected *Trichoderma reesei* Cel7A as a candidate and E1 a highly active endoglucanase - this mixture mimics the activities found in CelA) and CelA with a thermostable β-D-glucosidase to prevent end product inhibition^3^. The CelA blend was again tested at 75 °C and the Cel7A blend assayed at 50 °C. Effects of changes in cellulose crystallinity on the catalytic rate constant (*k*_s_) and in the first-order inactivation rate constant (*k*_i_) of the CelA and Cel7A blends were estimated using the kinetic model described above.

**Figure 2:**
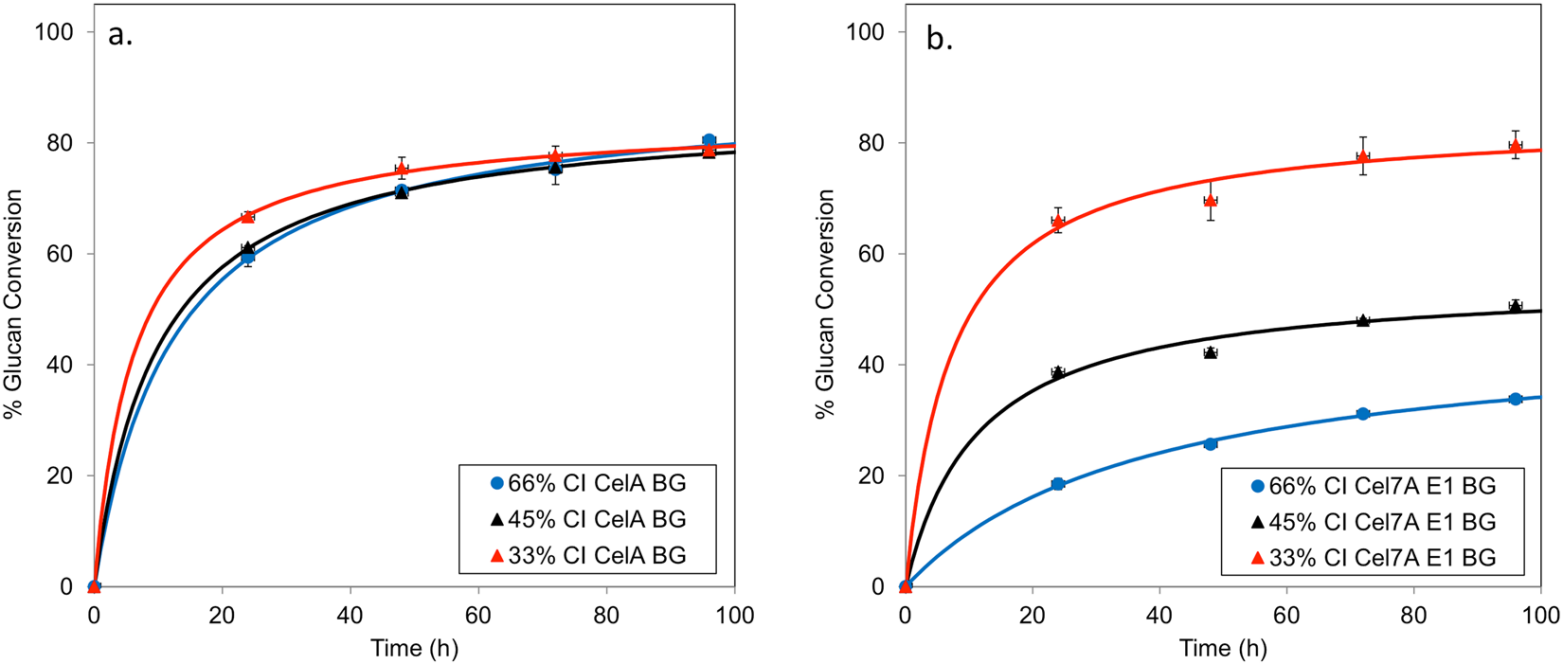
Digestion of differential crystal index (CI) materials by (**a**) mix of CelA and Bg and (**b**) by Cel7A, E1 and Bg indicate that while Cel7A is impacted significantly by CI, CelA is agnostic to CI of materials.

We found that both enzyme mixtures had similar performance on the low crystallinity (33%) substrate, but the fungal mixture had poorer performance on the high crystallinity (66%) substrate (Fig. 2b and Table 3). The value of *k*_s_ for the Cel7A blend on the most crystalline substrate was 6 h^−1^ (Table 3). This value increased 2.2 and 8-fold as the crystallinity index of the substrate decreased from 66% to 45 and 33%, respectively. However, cellulose crystallinity did not affect the cellulolytic activity of CelA. At a CI of 66%, *k*_s_ for the CelA-containing blend was 33 h^−1^. No significant change in *k*_s_ was observed when the CI decreased to 45%. At a CI of 33%, this value increased only modestly to 40 h^−1^. The extent of conversion for a high crystallinity substrate versus the low crystallinity substrate was nearly identical for all three levels of cellulose crystallinity (Fig. 2a and Table 3) using CelA. As expected, the *k*_i_ of CelA and Cel7A blends were unaffected by changes in the CI.

**Table 3:** Parameter values from model fits to CelA and Cel7A assay data conducted on cotton linters having different crystallinity indices (CI).

CI (%)	Enzyme	*k*_s_ (h^−1^)	K_L_ (g/L)	*k*_i_ (×10^−2^ h^−1^)	t_1/2_ (h)
33	CelA	40 ± 2	100^a^	2.5^b^	28
45	CelA	33 ± 1	100^a^	2.5^b^	28
66	CelA	33 ± 2	100^a^	2.5^b^	28
33	Cel7A	48 ± 6	100^a^	2.9^c^	24
45	Cel7A	13 ± 1	100^a^	2.9^c^	24
66	Cel7A	6 ± 1	100^a^	2.9^c^	24

The model was fit to each progress curve shown in Fig. S2 by varying *k*_i_ and *k*_s_. Best fit values ± standard deviations are shown.

^a^Value was fixed while estimating values for remaining parameters.

^b^Value was estimated using a global fit to CelA data all three substrates.

^c^Value was estimated using a global fit to Cel7A data on all three substrates.

These experiments demonstrate that cellulose crystallinity is not a performance-limiting property for CelA and that CelA is 5.5-fold more active on native cotton linters at its temperature optimum of 75 °C compared to Cel7A at its temperature optimum of 50 °C. Improvements in the performance of the Cel7A-containing blend were modeled as an increase in *k*_s_, while the value of the Michaelis-Menten constant, K_s_, was fixed. Indeed, the results could indicate that the specific activity of the bound fungal enzymes increase as the cellulose crystallinity index decreases. Alternatively, adsorption of the fungal cellulases may increase with decreasing cellulose crystallinity. In either case, this differs strikingly from the CelA-containing blend.

### CelA directly interacts with extracted lignin and is slightly inhibited by small molecular weight lignin

**Figure 3:**
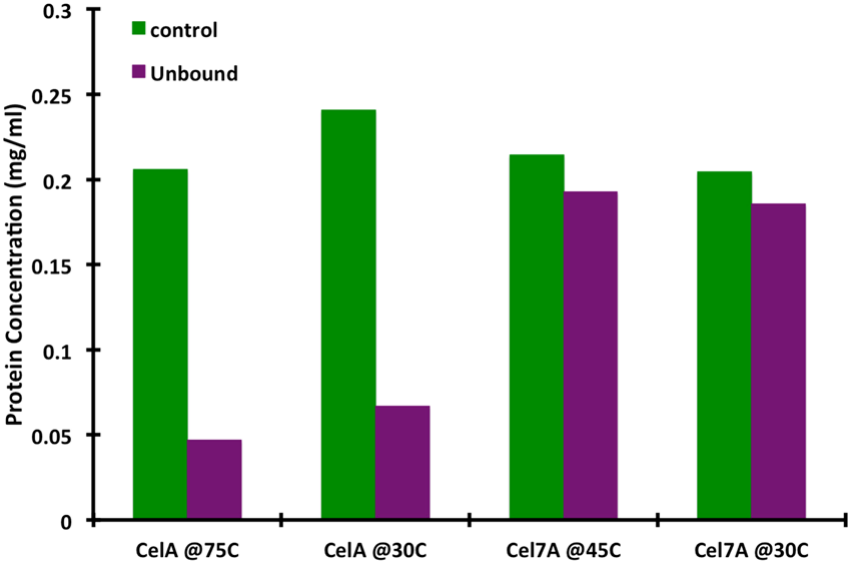
Protein concentration of CelA and Cel7A in the supernatant after incubation with insoluble lignin at 75 °C and 30 °C and at 45 °C and 30 °C, respectively, as measured by densitometry from SDS-Page Gel (Figure S6).

The putative negative effect of lignin was further explored by performing pull-down experiments using extracted lignin. CelA and Cel7A were incubated with lignin at 30 and their respective optimal temperatures of 75 and 45 °C for 1 h and then the bound and unbound pools were analyzed by SDS-PAGE. The Cel7A (Figure S6a) band intensity in the unbound fraction was similar to the control incubation done without lignin. In contrast, the CelA bands (Figure S6b) were substantially less intense in the unbound fraction relative to controls. This implies that Cel7A has a much lower affinity toward this extracted lignin at both temperatures tested compared to CelA. Densitometry was performed on these gels to quantify the amount of protein lost to lignin using Image J software^16^. The percent difference was calculated with CelA losing between 77% and 66% of the protein to interaction with lignin, whereas Cel7A lost only approximately 9%. Table 4 and Fig. 3 show the dramatic interaction of CelA with lignin at both temperatures.

**Table 4:** Protein concentrations with percent difference between the control and the unbound fraction.

	CelA (mg/mL)	CelA (mg/mL)	Cel7A (mg/mL)	Cel7A (mg/mL)
Temperature (°C)	75	30	45	30
Control	0.206	0.240	0.214	0.204
Unbound	0.047	0.067	0.193	0.186
Difference (%)	77.2	65.6	9.8	8.8

There is a 14.96% difference between 75 °C and 30 °C for CelA and a 10.09% difference between 45 °C and 30 °C for Cel7a.

**Figure 4:**
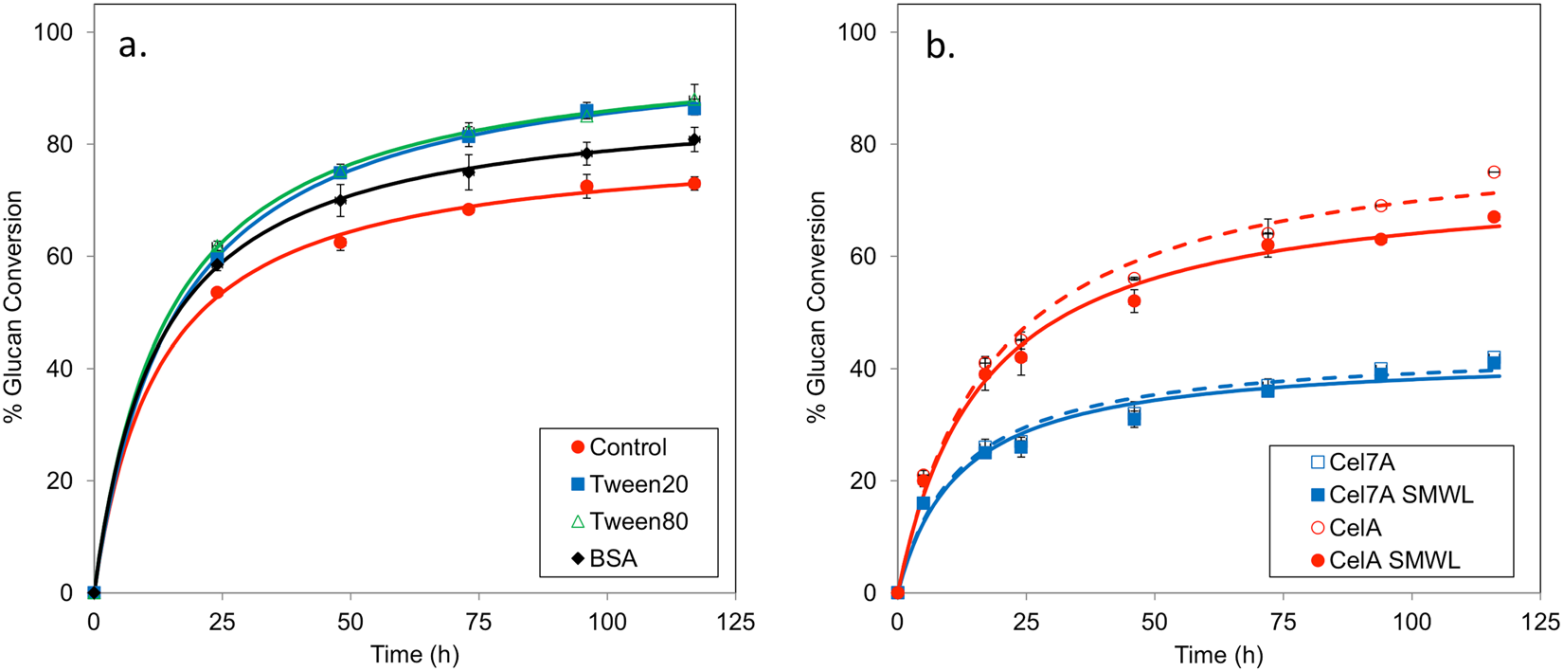
(**a**) Digestion of dilute acid treated corn stover by CelA is improved in the presence of surfactants (**b**) The effect of SMWL compounds decreases the performance of CelA on avicel.

Common surfactants, such as Tween 20 and Tween 80, and the serum protein, BSA, have been shown to improve biomass conversion presumably by blocking potential lignin binding sites^17–21^. We have utilized DA treated biomass to make samples rich in lignin and glucan, and performed enzyme digestions in the presence and absence of Tween 20/80 and BSA. These progress curves were modeled by varying K_L_ and *k*_i_. The data are shown in Fig. 4a and the model parameter values in Table 5. In the case of the Tween 20/80 treated samples, we observed a 15% increase in the extent of conversion at 117 h; whereas in the case of BSA, an 8% improvement in extent of conversion was noted at the same time (Fig. 4a). The CelA performance half-life increased significantly (p < 0.05, Student’s T-test) from 21 h for the control to 33 h and 31 h following addition of Tween 20 and Tween 80, respectively. The K_L_ increased modestly upon the addition of Tween 20 (1.9 g/L), Tween 80 (2.3 g/L) and BSA (2.1 g/L) relative to the control (1.6 g/L), however this difference was not statistically significant. Therefore, addition of Tween 20 and Tween 80 increased the performance half-life of CelA on DACS. This experiment provides further support that lignin represents a significant performance limitation for CelA.

**Table 5:** Kinetic parameters for CelA tested on DACS in the presence and absence of Tween 20, Tween 80 and BSA. The model was fit to each progress curve shown in Fig. 4 by varying *k*_i_ and K_L_. Values shown are best fit values and either their associated standard deviations or 95% confidence intervals.

Additive	*k*_s_ (h^−1^)	K_L_ (g/L)	*k*_i_ (×10^−2^ h^−1^)	t_1/2_ (h)	t_1/2_ 95% CI (h)
None	55^a^	1.6 ± 0.3	3.4	21	16–25
Tween 20	55^a^	1.9 ± 0.2	2.1	33^b^	28–39
Tween 80	55^a^	2.3 ± 0.2	2.3	31^b^	27–35
BSA	55^a^	2.1 ± 0.5	3.0	23	18–29

^a^Value was fixed while estimating values for remaining parameters.

^b^Value is significantly different than control tested in absence of additive (Student’s T-test, p < 0.05).

Other factors associated with the presence of lignin can also impact cellulase activity, such as inhibition by soluble low molecular weight lignin. To determine the effect of soluble lignin on the conversion of cellulose by CelA and Cel7A, enzymatic digestions were carried out on Avicel using both CelA and Cel7A in the presence of soluble small molecular weight lignin (SMWL). We found that there was on average a 9% decrease in the activity of CelA in the presence of SMWL, suggesting that SMWL has a small negative impact on CelA performance (Fig. 4b). Interestingly, there are no apparent differences in the digestion of cellulose by Cel7A, suggesting a tolerance for SMWL (Fig. 4b).

### CelA outperforms Cel7A in cellulose digestion even at a non-optimal temperature of 50 °C

**Figure 5:**
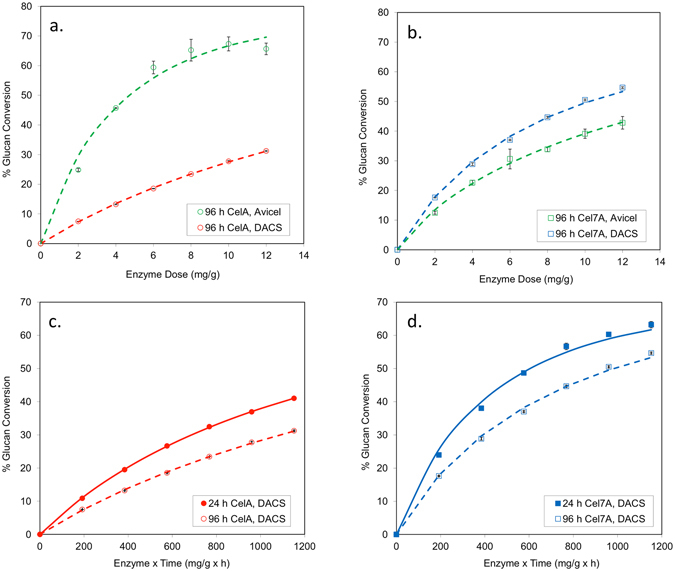
Performance of CelA and Cel7A on Avicel and DACS at 50 °C. CelA and Cel7A were incubated with Avicel and DACS at 50 °C for 24 and 96 h. β-glucosidase was added to each of these incubations at 5% total protein. Total enzyme doses ranged from 2 to 48 mg/g and glucose concentrations were measured by HPLC. (**a**,**b**) show the relative performance of CelA and Cel7A on these two substrates during 96 h incubations. This demonstrates that CelA is considerably better on Avicel than Cel7A even when tested at the temperature optimum of Cel7A. (**c** and **d**) show the 24 h and 96 h incubations for CelA and Cel7A, respectively, on DACS plotted as a function of enzyme x time. Model fits to these data are shown here – see Table 6 for parameter values. This demonstrates that the deviation in Et relationship is similar for CelA and Cel7A on DACS when tested at 50 °C.

The assays presented thus far were all conducted at 75 °C for CelA and 50 °C for CTec2 and Cel7A, their respective temperature optima. This difference in temperature optima is due in part to the higher melting temperature of CelA (80 °C) versus Cel7A (65 °C). Therefore is not clear whether the high activity of CelA on Avicel compared to CTec2 and on native cotton linters compared to Cel7A is principally due to differences in its catalytic mechanism or due to Arrhenius effects on catalytic rate. To address this question, CelA and Cel7A were tested on Avicel and DACS at 50 °C for 96 h using different enzyme loadings ranging from 2–12 mg/g. These results are shown in Fig. 5.

Interestingly, even at 50 °C the performance of CelA is markedly higher than Cel7A on Avicel. This indicates that Arrhenius effects alone do not account for the remarkable activity of CelA on pure cellulose and that the intrinsic catalytic mechanism of CelA is particularly effective in degrading this substrate. Another observation from earlier experiments was that both CelA and fungal enzyme systems exhibited apparent first-order inactivation effects, captured in the model fits by *k*_i_. Given that these enzymes were tested over the course of up to five days, it is plausible that inactivation of CelA at 75 °C and CTec2/Cel7A at 50 °C limits their hydrolysis performance. However, the magnitude of *k*_i_ varied considerably for CTec2 on different lignocellulosic substrates implying that thermal inactivation is not the only factor contributing to the apparent inactivation rate.

To investigate this further, CelA and Cel7A were tested on DACS at 50 °C. Two different ranges of enzyme dilutions were used. Enzyme doses ranging from 8 to 48 mg/g were incubated with DACS for 24 h while 4-fold lower doses of enzyme were incubated with substrate for 96 h. This experimental set-up yields two dose-response profiles for each enzyme, with different incubation times but equivalent sets of enzyme × time (Et) values. These results are shown in Fig. 5c,d plotted as a function of enzyme × time. If enzyme performance is entirely stable over time then the 24 h and 96 h profiles would superimpose whereas any deviation in the Et relationship where the 96 h profile falls below would be consistent with time-dependent inactivation of the enzyme^22^. These results were also fit to the two-stage kinetic model and the estimated parameter values are shown in Table 6.

**Table 6:** Parameter values for model fits to CelA and Cel7A Et curves on DACS.

Enzyme	*k*_s_ (h^−1^)	*k*_i_ (×10^−2^ h^−1^)	t_1/2_ (h)	t_1/2_ 95% CI (h)
CelA	5 ± 1	1.5	48	44–52
Cel7A	15 ± 1^a^	1.6	44	35–61

Values shown are best fit values and either their associated standard deviations or 95% confidence intervals.

^a^Value is significantly different than CelA (Student’s T-test, p < 0.05).

Strikingly, the deviations in the Et relationship shown in Fig. 5 for CelA and Cel7A are very similar. The model fits to these data indicated that the t_1/2_ of CelA was 48 h under these conditions while that for Cel7A was 44 h. One would expect that CelA, having a substantially higher T_m_ would be more stable than Cel7A and exhibit little-to-no deviation in Et relationship at 50 °C. Therefore, the parameter *k*_i_ may not exclusively reflect the rate with which CelA transitions from a native to a denatured and inactivated form over time. Perhaps limitations imposed by an insoluble substrate and the need to alternately adsorb and desorb from the cellulose surface to access sites susceptible to enzymatic activity also contribute to deviations in the Et relationship and *k*_i_. If so, then the actual stability of CelA in hydrolysis at 75 °C is greater than that implied by the half-lives reported here.

### Physical substrate analyses of biomass at the particle and tissue scale reveal the differential impact of pretreatment chemistry

**Figure 6:**
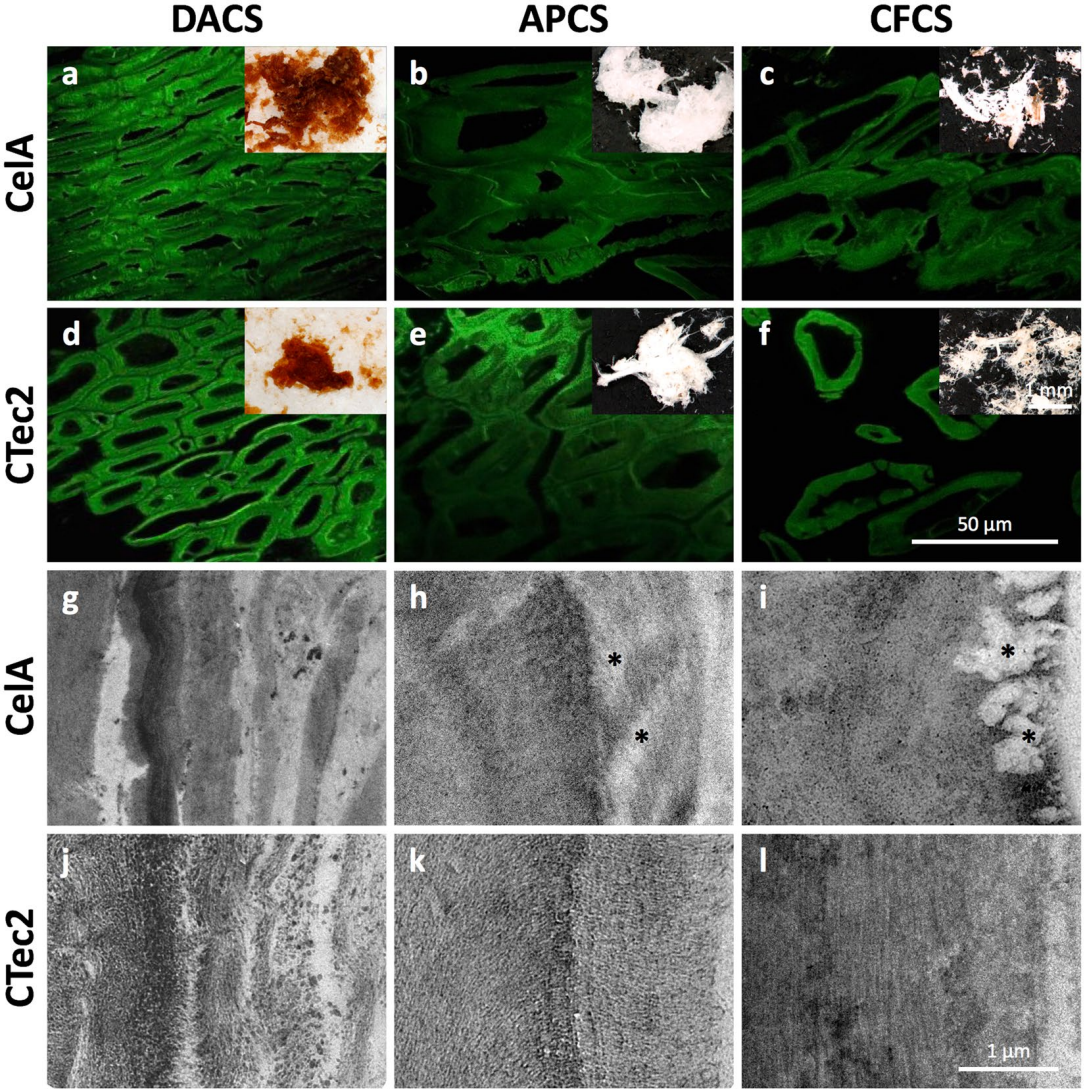
(**a**–**f**) CSLM micrographs (with stereoscope micrograph insets) of digested corn stover particles display morphological features typical of materials exposed to DA, AP, and CF pretreatments. Among these, the clearest evidence for cellular dislocation and deconstruction at the tissue scale is seen in the samples exposed to CF pretreatment (**c**,**f**). At this scale, there are no clear or consistent differences between the CTec2 and CelA digested samples (**g**–**l**). TEM micrographs of secondary cell walls from fiber cells show evidence for delamination, wall loosening and enzymatic deconstruction. The DACS micrographs show the coalescence and relocalization of lignin into globules that is typical of dilute acid (**g**,**j**) pretreatment. The APCS cell walls (**h**,**k**) show lower contrast and reveal cellulose structure due to lignin removal and show evidence for channels or cavities formed in CelA digested material (_*_ **k**). The digested CFCS samples (**i**,**l**) display the clearest evidence for a difference between the deconstruction mechanism of CelA compared to CTec2 revealing completely cleared cavities near the surface of cell wall regions (_*_ **i**) similar to the cavities previously observed in Avicel particles.

The most dramatic difference between the pretreated substrates visible in the stereo micrographs (Fig. 6a–f insets) is the color of the biomass particles. The dilute acid pretreated corn stover (DACS) material appears orange-brown in color, indicative of lignin re-localization to the particle surface and of cell wall carbohydrates undergoing Maillard reactions. In contrast, both the alkaline peroxide corn stover (APCS) and clean fractionation corn stover (CFCS) samples appear mostly white, evidence of essentially complete lignin removal. The clean fractionation samples are more fiberized, less clumped and display mostly individually separated fiber cells or small bundles of fibers (Fig. 6c,f insets). For each pretreatment, the CelA and CTec2 digested residues appear largely similar at this particle scale and comparing them provides no clues into differences in deconstruction mechanisms.

To investigate tissue and cellular scale differences among the samples, we sectioned the treated corn stover particles in preparation for examination by confocal scanning laser microscopy (CSLM) (Fig. 6a–f). Like the stereo micrographs, the CSLM micrographs revealed the morphological features typical of materials exposed to DA, AP, and CF pretreatments^23–25^. Whereas all three pretreatments exhibited some evidence for cell-cell dislocation (Fig. 6a–c), the clean fractionation samples are extreme in this regard with many areas of tissue appearing devoid of remaining middle lamella. Like the particle-scale analysis, there were no distinguishing features between the CTec2 and CelA digested materials to provide insight into differences in deconstruction mechanisms either between the two enzyme systems or between the differently pretreated substrates.

At the scale of cell wall architecture, transmission electron microscopy (TEM) images show the coalescence and delocalization of lignin into globules, which is typical of DA pretreatment (Fig. 6g,j)^26^. Both the CTec2 and CelA enzyme systems appear to have penetrated into at least portions of these cell walls as evidenced by the lower density cell wall zones proximal to the cell lumen^27^. The digested, AP-pretreated cell walls showed low contrast and revealed the clearest evidence of the oriented cellulose structure and enzymatic activity likely due to extensive lignin removal. The digested, CF-pretreated samples displayed the clearest difference in morphological evidence for enzymatic activity, with the CelA-digested samples revealing cleared cavities near the surface of some cell wall regions (Fig. 6i) similar to the cavities previously observed in Avicel PH-101 cellulose particles^3^.

### Immuno localization of the CelA enzymes confirms their presence in the cavities and suggests that enzyme generated accessibility explains differences in deconstruction

**Figure 7:**
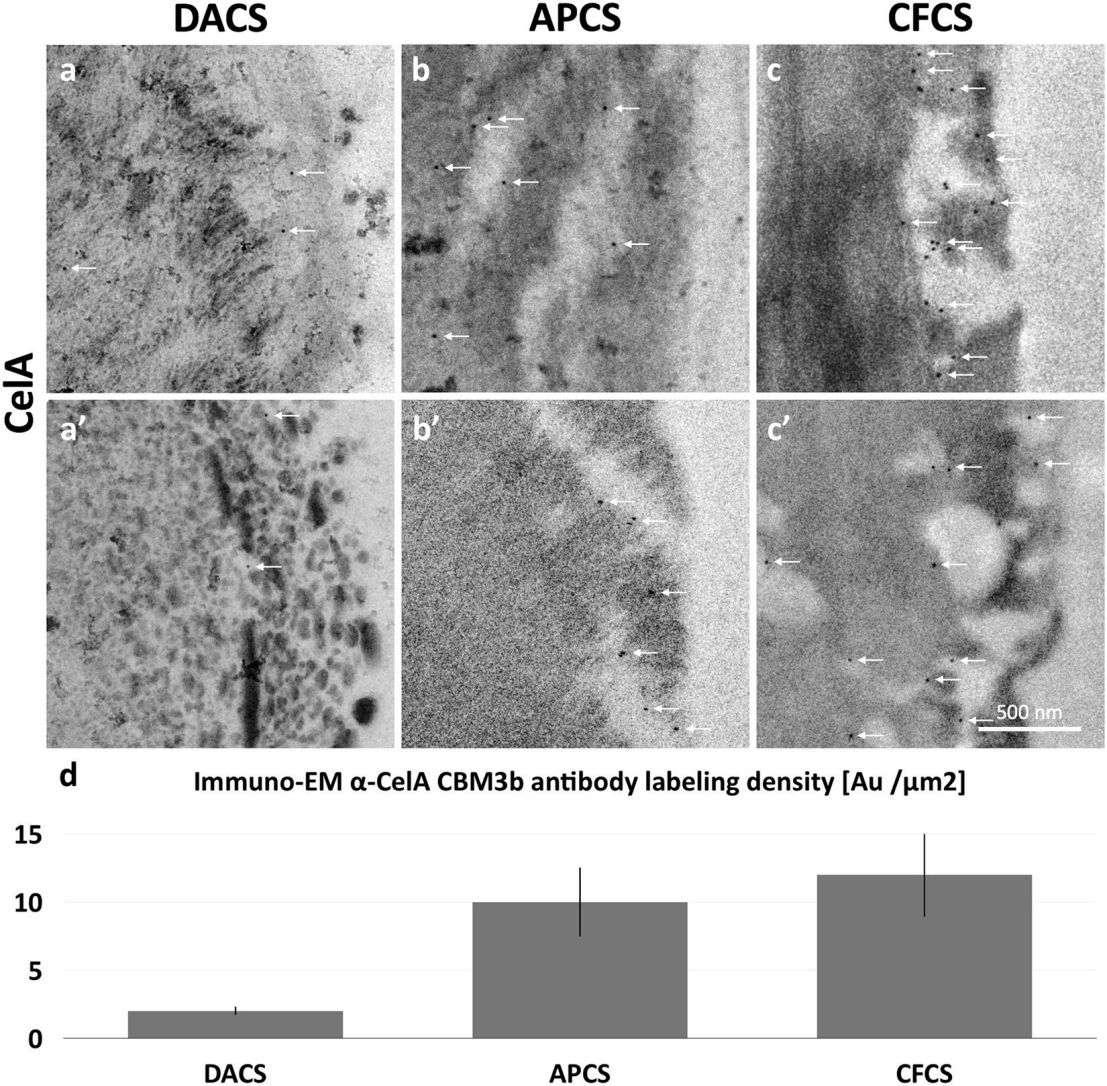
Immuno-EM micrographs reveal the pattern of penetration of CelA enzymes into pretreated corn stover cell walls and confirm that CelA enzymes occupy the cavities generated in APCS and CFCS. (**a**,**a’**) The labeled enzymes (white arrows) in DACS samples appear in cleared zones well into the secondary cell wall and do not appear to be associated with surface lignin globules. (**b**,**b’**,**c**,**c’**) CelA enzyme labeled in APCS and CFCS cell walls were most often found within cavities that connect to the cell wall surface and penetrate into the secondary cell wall. (**d**) Quantitation of the immune gold α-CelA CBM3 labeling density shows 3–4 times the labeling density in the digested APCS or CFCS compared to DACS.

To determine if the cavities seen in the CelA digested biomass cell walls were indeed created by the activity of these enzymes, we performed immuno-EM to localize the CelA enzymes in the substrate. The labeled enzymes (white arrows) in DA-pretreated samples (Fig. 7a,a’) appear in cleared zones well into the secondary cell wall, not only on the luminal cell wall surface and do not appear to be associated with surface attached lignin globules. This finding indicates that at least some of the CelA enzyme is capable of navigating the maze of relocalized lignin at the cell wall surface. CelA enzyme labeled in AP-pretreated and CF-pretreated cell walls were usually found within cavities that connected to the cell wall surface and penetrated deep into the secondary cell wall. This observation suggests that cavity formation is a prevailing strategy that CelA employs to digest biomass. Quantitation of the immuno-EM labeling shows that 3 to 4 times as many CelA enzymes bind the lignin-extracted substrates as to the DA pretreated material (Fig. 7d).

## Discussion

Minimally augmented CelA based mixes were much more efficient at converting biomass on a molar basis when compared to optimized fungal cellulase mixtures acting on all substrates tested. However, these greatly different cellulase systems are roughly equivalent when compared on a total protein mass loading basis, which is the industry’s standard used to calculate cost^3^.

*C. bescii* based enzyme mixtures have a strong preference for de-lignified substrates when compared to a dilute acid treated substrate (Fig. 1). Furthermore, when lignin-rich DA treated corn stover is digested in the presence of surfactants known to block lignin association by enzymes, we observed a significant (15%) improvement in the overall extent of conversion of the DA treated material (Fig. 4). This observation suggests a possible inhibitory mechanism for CelA that may be distinct from free fungal enzymes that have minimal affinity for some lignins (Figs 3 and 4b). This propensity for unproductive binding, coupled with a reduction in the critical molar loading of CelA compared to free enzymes (Cel7A), could explain the lower performance demonstrated by CelA on lignin rich substrates. In essence, CelA cannot convert cellulose to soluble sugars while it is bound to lignin, and on an equal mass-loading basis this impact is far more severe when compared to the smaller fungal cellulases. Understanding the specific sites of this lignin-CelA interaction and finding ways to minimize these interactions while preserving cellulose binding would be of great importance to the biofuels industry.

Lignin has been reported previously to negatively affect enzyme performance by binding cellulases non-productively in a reversible manner and contributing to irreversible enzyme inactivation^28^. For this reason, a kinetic model was used to determine whether differences in CelA performance were due to changes in the initial rate of reaction or to changes that were time-dependent. This provides additional insight on the nature of the challenges lignin poses towards CelA and how they may be different from canonical fungal cellulase systems. While Michaelis-Menten-based models similar to that described here have been used previously for modeling biomass conversion, there are clearly potential limitations in applying them to cellulose degradation. For example, cellulose could become increasingly recalcitrant in a conversion dependent manner. Drissen *et al*.^29^ reported and proposed a model for such a phenomenon. However, this subject remains controversial. Desai *et al*.^30^ concluded that this effect is minimal. Similarly, Hall *et al*.^13^ show that the crystallinity of Avicel remains unchanged throughout hydrolysis. In addition, a Langmuir-like dependence of initial glucose production rate as a function of cellulase dose has been reported by others^29, 31^ indicating that cellulose accessibility can be limiting to the rate of reaction, depending on the enzyme dose. Scott *et al*.^32^ tested a fungal cellulase mixture on pretreated wheat straw using enzyme doses similar to those used here and observed no evidence of either increasing substrate recalcitrance or enzyme saturation. One would anticipate that properties such as enzyme binding capacity or increasing recalcitrance would be highly substrate dependent. However, the CelA data shown here on APCS, CFCS and Avicel, were well modeled using the same inactivation rate constant and very similar catalytic rate constants. This implies that the substrates are remarkably similar with respect to CelA activity and if these substrates either became increasingly recalcitrant to CelA as a function of conversion or were in any case saturated with enzyme, this was remarkably similar given the differences in how these substrates were prepared.

A second, unprecedented, finding of this study is that for traditional fungal enzyme systems, the crystalline nature of cellulose has been previously described as a significant barrier^10, 12, 33^. Indeed, our experiments confirm that for fungal Cel7A, augmented with the E1 endoglucanase (used to mimic the bifunctional nature of CelA), increasing cellulose crystallinity significantly impedes the rate and extent of digestion (Fig. 2b). However, for CelA, the crystallinity of the material seems to have no impact on the rate of digestion or the actual extent of conversion (Fig. 2a) [Note that the molar loading of CelA is approximately four-fold lower than the equivalent Cel7A mixture]. To our knowledge, no other known cellulase system displays such disregard for cellulose crystallinity - long discussed as one of the key recalcitrance barriers^10, 13^. For practical applications, it is known that both populus and loblolly pine, two candidates for industrial biomass substrates, have crystallinities of approximately 63% and are difficult for fungal cellulase cocktails to convert compared to other biomass substrates, such as switchgrass or corn stover which have significantly lower crystallinity^34^.

Our initial findings for Avicel digestions suggested that CelA can excavate cavities into biomass, we wanted to confirm that this was mechanistically correct on more relevant pretreated biomass substrates^35^. Based on TEM imaging shown for three different pretreatments, we do in fact observe the formation of cavities into plant cell walls in all cases (Figs 6 and 7). Furthermore, TEM antibody labeling of CelA does seem to trend with the extent of conversion observed. The total number of CelA molecules detected by TEM-immunolabeling is the highest in the clean fractionated samples, second highest in the AP treated biomass, and significantly lower in the DACS treated material (Fig. 7). Given that the lignin content of the DACS sample is much higher than the other samples; as well as data from the SDS-PAGE experiments, we have determined that CelA adsorbs to lignin and conclude that CelA molecules are likely non-productively associated to lignin-rich regions of this substrate.

CelA continues to be a fascinating enzyme, and is indeed the first enzyme shown to be insensitive to substrates crystallinity. Additionally, it is competitive with fungal enzymes at one-fourth the molar enzyme loading when acting on a variety of pretreated substrates. However, CelA is most active on delignified substrates. Finally, the cavity forming mechanism of CelA is unprecedented and now reproduced on biomass substrates. This further elucidation of the mechanism of action of this enzyme suggests that enzyme preparations could be engineered to take advantage of the synergy employed by CelA and extended to heterologous CAZYmes forming new multi-functional cellulases.

## Materials and Methods

### Biomass pretreatments

#### Alkaline peroxide (AP) pretreatment

Alkaline peroxide (AP) pretreatment was performed on raw corn stover (CS) in a 2 L shake flask according to Selig *et al*.^36^. A 30% (w/w) stock solution of hydrogen peroxide was added at 500 mg H_2_O_2_/g CS followed by the addition of deionized water to obtain a solids loading of 10%. The pH of the suspension was adjusted to pH 11.5 by adding 5 M NaOH. AP pretreatment was performed in a shake incubator for 48 h maintained at room temperature (25 °C). No additional NaOH was added during the course of the pretreatment. After 48 h, the pretreated suspension was filtered through a coarse felt, and solids fraction was thoroughly washed with deionized water to neutral pH and stored at 4 °C.

#### Clean Fractionation (CF) pretreatment

CF pretreatment was performed using a single-phase mixture of water and acetone in combination with methyl isobutyl ketone (MIBK) in the following ratio: MIBK/acetone/water (11/44/44, g/g/g, 100 mL) with sulfuric acid concentration at 1.2 wt%^37^. Whole corn stover was loaded in a 316 stainless steel pressure reactor at 10% solids. The reactor was sealed and heated in a sand bath at 160°C for 45 min. After the pretreatment, the reactor was cooled in ice water. The pretreated suspension was separated into a solid fraction and an aqueous fraction via filtration. The solid fraction was thoroughly washed first with the same solvent (250 mL) followed by deionized water to neutral pH and stored at 4 °C.

#### Deacetylated dilute acid (DA) pretreatment

Corn stover biomass was prepared and pretreated as follows: whole stover was pretreated in the 200-kg/day continuous, high-solids, pilot-scale horizontal pretreatment reactor system at NREL using 2% sulfuric acid at 158 °C with a residence time of 5 min. Deacetylation of the stover was performed using 0.1 M NaOH at an 8% solids loading at 80 °C, and mixed at 15 rpm for 2 h prior to dilute-acid pretreatment. The pretreated material was washed with water by centrifugation until a pH of 5 was observed. The biomass was analyzed as previously described^38^.

### Generation of cellulose at varying degrees of crystallinity

Amorphous cellulose and cellulose-I at varying degrees of crystallinity were prepared from cotton linters (CAS Number 9004-34-6; catalogue number 22183) obtained from Sigma-Aldrich by using a method described by Hall *et al*.^13^. Briefly, 1 g of dry cellulose powder was added to 30 mL of ice-cold concentrated phosphoric acid. The slurry was allowed to react at 0 °C for 40 min with occasional stirring. After 40 min, 20 mL of ice-cold acetone was added to the slurry followed by stirring and filtration on a sintered glass crucible. The filtered sample was further washed three times each with 20 ml of ice-cold acetone and DI water. The resulting cellulose was freeze-dried. The cellulose-I samples at reduced crystallinity (PASC-CL, CI-44 and PASC-CL, CI-34) were prepared by varying the phosphoric acid concentration using the same method describe above.

### X-ray diffraction measurements

The crystallinity indexes (CI) of cellulose samples were measured by X-ray diffraction (XRD) by using a Rigaku (Tokyo, Japan) Ultima IV diffractometer with CuKα radiation having a wavelength λ(Kα1) = 0.15406 nm generated at 40 kV and 44 mA. The diffraction intensities of dried samples placed on a quartz substrate were measured in the range of 8 to 42° 2θ using a step size of 0.02° at a rate of 2° min-1. The crystallinity indexes of the cellulose samples were measured according to the amorphous subtraction method described by Park *et al*.^39^. A diffractogram of amorphous cotton linter cellulose sample mentioned above was subtracted from the other cellulose samples to remove the influence of the amorphous component in the diffractograms. The ratio of the integrated area of each subtracted diffractogram to the area of the original was then calculated and multiplied by 100 to give the CI value of the sample.

### Compositional analysis of pretreated solids

To determine the structural carbohydrates and lignin components of the pretreated solids, the compositional analysis was conducted according to the NREL method^38^.

#### Lignin extraction method

Steam explosion pretreatment of corn stover - Pretreatment of corn stover was conducted in the NREL four-L steam explosion reactor at 180 °C, 1 wt% H_2_SO_4_, for 3 min^40^. The reactor is constructed of Hastelloy C-22 for corrosion resistance. A two-inch thick insulating jacket surrounds the steam jacket and temperature controlled electrical heating bands that encase all external surfaces of the reactor, limiting heat loss to the environment, and reducing condensation inside the reactor during pretreatment. The pre-warmed reactor was loaded with 500 g of acid impregnated and pressed corn stover (~43% solids), sealed with the top ball valve, and steam applied to both the top and bottom of the reactor interior to quickly heat (~5 to 10 s) the biomass to reaction temperature. The timer is started when the reactor contents measured by two thermocouples inside the reactor reach reaction temperature. The bottom ball valve is quickly opened at the desired experimental residence time and the pretreated solids are blown into a nylon HotFill® bag inside a 200-L flash tank. The bag is removed from the flash tank, labeled, sealed, and stored at 4 °C until ready for analysis. This allows collection of all steam and volatile components (furfural and acetic acid) in the slurry for more accurate component mass balance measurements.

### Lignin extraction method

The pretreated corn stover was extracted with aqueous dioxane utilizing a modified Bjorkman method where by the milling and 0.1 M HCl reflux at 90 °C steps have been eliminated as the samples have already been milled and pretreated under acidic conditions^41^. Approximately 100 g wet weight samples of pretreated solid residues were washed five times with DI water to remove all soluble carbohydrates and by products that may have been generated during the pretreatment process. The washing process involves suspension of the sample solids in 100 mL of DI water and filtered using 90 mm Whatman glass fiber (GF/A) filters in appropriate sized Buchner funnels, then re-suspended in DI water for the next round of filtration. The water washed solids were then suspended in 600 mL of a 9:1 (v/v) mixture of dioxane (1,4-dioxane, J.T. Baker) and DI water and extracted for 1 h at 120^o^C with intermittent stirring to keep the solid particles suspended. The dioxane extracted solids were then filtered over Buchner funnels equipped with Whatman GF/A glass fiber filters to separate the extracted solid residues from the liquor filtrates. The extracted solids were washed with 500 mL of 95% ethanol followed by a 500 mL wash with DI water.

The soluble solids within the dioxane:water extraction liquors were concentrated to 50 to 100 mL utilizing a rotary type evaporator, then precipitated by adding cold DI water (4X the final extract volume), and centrifuged in a GSA rotor at 9000 rpm for 30 min. The precipitated solids were washed 3x with 150 mL DI water and freeze dried for characterization by NMR, GPC, and Raman spectroscopy, the results of which indicate minor changes to the lignin molecular weight with increasing pretreatment temperature (data not shown). The yield of lignin extracted with 1:9 dioxane was performed, which ranged from roughly 15% to nearly 40% of the resident lignin content by weight.

### Cellulase purification

#### CelA purification

A hydrophobic affinity purified *C. bescii* broth enriched in CelA was utilized as described in refs 35, 42 for the CelA mix data reported here, we emphasize that this is not the raw *C. bescii* exproteome, rather it is enriched in the hydrophobic components such as CelA that represents a the majority of the activity of the broth^2^. For the experiments requiring pure CelA, CelA was tagged with 6xHis tag, and expressed in *C. bescii*. CelA was purified out of the *C. bescii* exoproteome using a 5 mL HisTrap fast flow column (GE) and was further purified using a Superdex 26/60 200 PG column.

*T. maratima* β-D-glucosidase and β-xylanase were purchased from Megazyme (Bray, Ireland) samples were desalted using a Hi-trap 26/10 (GE life sciences) desalting column before use to remove ammonium sulfate stabilizer.

E1 was purified as described previously^3^.

#### Cel7A Production and purification

The transformed *Trichoderma reesei* cell culture was streaked on a Potato Dextrose Agar plate and allowed to grow 2–3 days until a well lawned plate of spores was achieved. A ~0.5 cm plug was extracted from the plate and deposited into 1 L of liquid growth media in a 2.8 L shake flask. The growth media consisted of Mandel’s Growth Media with 5% glucose as the carbon source, and 0.5% tryptone added. The culture was grown at 28 °C with agitation for 24 hours, after which the entire 1 L was transferred to 7 L of the same media, in a bioreactor. The bioreactors were 15 L working volume vessels manufactured by New Brunswick and controlled via New Brunswick’s BioFlo310 system. The total of 8 L was grown with mixing at 300 rpm via dual down-flow marine style impellers, purged with 1.5 VVM of filtered air, kept at a strict 28 °C, and pH controlled at 4.8. The acid and base used for pH control was HCl and KOH, respectively. The cell culture was grown for 48 hours, after which the entire culture broth was drained, filtered through nylon to remove all cell mass, and concentrated via tangential flow filtration with a 10,000 Dalton MWCO (GE Health Sciences). The concentrated broth was buffer exchanged into 20 mM Bis-Tris pH 6.5, and brought up to ~200 ml.

Fermentation broths (~8 to 10 L) were harvested and sequentially vacuum filtered through the following series: (1) Miracloth (EMD Biosciences), (2) ~2 µM glass fiber filter, (3) 1.1 µM glass fiber, and (4) a 0.45 µM PES membrane. This filtered broth was then concentrated by tangential ultrafiltration with a 10,000 Da MWCO. Broths were roughly concentrated from 8 L to 150 mL. This volume was exchanged with at least 1 L of 20 mM Bis-Tris pH 6.5 buffer to remove residual peptides and other low molecular weight debris. This concentrate was then re-filtered to 0.2 µM and the filtrate was adjusted to 1.5 M (NH_4_)_2_SO_4_ for hydrophobic interaction chromatography (HIC) and then loaded onto a 26/10 Phenyl Sepharose Fast Flow column. For HIC, buffer (A) is 20 mM Bis-Tris pH 6.5 and buffer (B) is 20 mM Bis-Tris pH 6.5, 2 M (NH_4_)_2_SO_4_. Buffers were run 80% B to 0 over 8 column volumes. Active fractions are identified by a *p*NP-lactose (*p*NPL) activity assay (*p*NPL at 2 mM in 50 mM acetate pH 5.0.) One hundred µL of *p*NPL added to each well of a 96-well plate. Twenty five µL of each fraction were added and the plate incubated 30 min at 45 °C. Reactions were quenched with 25 µL of 1 M NaCO_3_ and the absorbance at 405 nm (A_405_) was measured. Standard curve concentrations ranged from 0 to 250 µM *p*NP.

*p*NPL-active fractions were pooled and concentrated as needed. Protein was desalted and exchanged into 20 mM Bis-Tris pH 6.5 buffer. We next utilized a 10/100 anion exchange column packed with Source 15Q run at 0 to 50% 30 cv, the buffers were 20 mM Bis-Tris pH 6.5 and same buffer + 1 M NaCl. *p*NP-lactose activity was followed again to identify active fractions. SDS-PAGE and αCel7A immunoblotting (described elsewhere) was performed to assess purity. The final stage of purification consisted of size exclusion chromatography (SEC) using 26/60 Superdex 75 column and 20 mM acetate pH 5.0 buffer containing 100 mM NaCl.

For single antibody Western blots immuno-detection of Cel7A was achieved using the SNAP i.d. Protein Detection System (Millipore Corp., Billerica MA). The PVDF membrane was blocked using SuperBlock PBS (Thermo Fisher Scientific Inc., Rockford, IL) for 20 min. Rabbit anti-Cel7A polyclonal IgG was used as the primary antibody (1:20,000 dilution of crude serum), with alkaline phosphatase-conjugated goat anti-rabbit IgG (Thermo Fisher Scientific Inc., Rockford, IL) as secondary. The alkaline phosphatase localization was visualized using BCIP/NBT (Life Technologies Corp., Carlsbad, CA).

### SDS-PAGE gel assays

Pre-cast 4–12% SDS-PAGE gels (Life Technologies, Carlsbad, CA) for Cel7A and for CelA were used to visualize proteins bound and unbound to lignin extracted from corn stover. All gels were run at 200 V constant for 50 min in MOPS-SDS buffer. For binding studies, 300 μg of desalted protein was incubated with 6 mg of lignin, corresponding to a process loading of ~30 mg protein/g cellulose for a theoretical biomass containing 30% lignin and 50% cellulose. The protein/lignin combination was incubated at room temperature for 60 min in 25 mM sodium citrate buffer at pH 4.8 unless otherwise stated. After the incubation, the lignin was centrifuged at 20,000 × g for five min and the supernatant containing the unbound protein was collected while the lignin pellet was washed an addition four times with citrate buffer. The desalted starting enzyme, supernatant containing unbound proteins, and the lignin pellet containing protein bound to insoluble lignin were diluted with 4X LDS sample buffer (3:1 sample:buffer) and held at 80 °C for 10 min in preparation for SDS-PAGE.

### Alternatively Pretreated Substrates Enzyme Digestions

Differentially pretreated substrates were digested at a total enzyme loading of 15 mg/g glucan. For CelA, digestions we utilized 11.5 mg/g of CelA broth, 3 mg/g E1, and 0.5 mg/g β-D-glucosidase as discussed in the enzyme purification section. For the second digestion set, the loadings for DACS and APCS were 10 mg/g CelA broth, 2 mg/g E1, 2 mg/g β-D-xylosidase and 0.5 mg/g β-D-glucosidase. For the CFCS substrate, it was necessary to lower the total loading to 5 mg/g glucan the composition in this case was: 4.25 mg/g glucan, 0.5 mg/g E1 and 0.25 mg/g beta glucosidase. CTec2 from Novozymes was used as a model fungal free enzyme system and was loaded at either 15 mg/g in all cases or 5 mg/g glucan for the CFCS substrate. CelA mix digestions were performed at 75 ^o^C at pH 5.5 while the CTec2 based digestions were run at 55^o^C pH 5.0. All digestions were conducted at a total initial solids loading of 1%.

Digestions were run continuously for 5 days with sampling at various time points. Enzymes were inactivated by boiling for 15 min after which samples were filtered through 0.45 mm Acrodisc syringe filters. The released sugars were analyzed by HPLC. Samples were injected at 20 μL volume and run on an Agilent 1100 HPLC system equipped with a BioRad Aminex HPX-87H 300 mm × 7.8 mm column heated to 55 °C. A constant flow of 0.6 mL/min was used with 0.1 M H_2_SO_4_ in water as the mobile phase to give optimal sugar separation. Glucose, xylose, cellobiose and xylobiose were quantified against independent standard curves and converted to anhydrous glucan equivalent and the results are reported as anhydrous glucan converted. All experiments were performed in triplicate and the resulting extents of conversion are shown as percent glucan or xylan converted.

### Differential Crystallinity cellulose digestions

Three differential crystallinity substrates were digested with CelA complemented with a *T. maritima* β-D-glucosidase. Total protein loading was 14.5 mg/g CelA and 0.5 mg/g β-D-glucosidase. Experiments were run in triplicate at 80 °C. Cel7A from *T. reesei* and E1 from *A. cellulolyticus* were loaded at 7.25 mg/g each and 0.5 mg/g β-D-glucosidase. Experiments were run in triplicate at 50 °C. All digestions were conducted at a total initial solids loading of 1%. Digestions were run continuously for 5 days with sampling at various time points. Enzymes were inactivated by boiling for 15 min after which samples were filtered through 0.45 mm Acrodisc syringe filters. The released sugars were analyzed by HPLC following the protocol described above.

### Surfactant assisted biomass digestions

DACS corn stover was digested with 10 mg/g *C. bescii* broth, 4 mg/g E1 and 0.5 mg/g b-D-glucosidase in the presence or absence of Tween 20 (0.4%), Tween 80 (0.4%) and 0.04% BSA. Experiments were run in triplicate at 80^o^C. All digestions were conducted at a total initial solids loading of 1%. Digestions were run continuously for five days with sampling at various time points. Enzymes were inactivated by boiling for 15 min after which samples were filtered through 0.45 mm Acrodisc syringe filters. The released sugars were analyzed by HPLC following the protocol described above.

### Hydrolysis of DACS and Avicel PH101 by CelA or Cel7A

Dose-response experiments of CelA and Cel7A were carried out in 96-well plates (Fisher Scientific) at 2% total solids substrate loading at 50 °C, pH 5.0, equivalent to 1% initial cellulose. Each of these enzymes was blended with β-glucosidase at a protein mass ratio of 95% CelA or Cel7A:5% β-glucosidase. Two sets of enzyme loadings were used for testing on DACS. The loadings used for 24 h incubations (8–48 mg/g cellulose) were 4× relative to the loadings used for 96 h incubations (2–12 mg total protein/g cellulose). The total volume of the saccharification slurries after adding enzyme and 50 mM acetate buffer, pH 5.0 was 0.25 mL. These incubations were done with end-over-end mixing at 12 rpm in a Fisher Isotemp incubator for 24 and 96 h. To measure cellulose conversion, 200 μL of each hydrolysate sample was filtered using a 0.45 µm 96-well filter plate (Millipore) and diluted 2-fold in 5 mM H_2_SO_4_. Glucose concentrations were measured by HPLC (Agilent) using an Aminex HPX-87H column (BioRad Laboratories) using a 5 mM sulfuric acid mobile phase and a flow rate of 0.6 mL/min. The sample injection volume was 20 µL and the run time was 11 minutes. The glucose concentration from each reaction was divided by the maximal glucose yield, obtained by loading an excess amount of enzyme, in order to calculate a fractional glucan conversion for each reaction.

CelA and Cel7a were also tested on Avicel PH101 under similar conditions described for the 96 h incubations above, except that the total initial solids loading as 1%.

### Kinetic Modelling

Enzyme mixtures comprising *Caldicellulosiruptor bescii* CelA, Cel7A, or CTec2 cellulose hydrolysis progress curves were analyzed using the two-stage kinetic model shown in Fig. S1. The first stage in the model involves the conversion of cellulose to cellobiose (panel a). As described, all of the components except for β-D-glucosidase, contributed to the formation of cellobiose. Similarly, only β-D-glucosidase catalyzed the conversion of cellobiose to glucose (panel b). Both stages were assumed to follow Michaelis-Menten kinetics. It was also assumed that the cellulases in Stage 1 were subject to competitive inhibition by lignin (K_L_), glucose (K_G_^a^) and cellobiose (K_G2_) and subject to first-order inactivation (*k*_i_) to the inactive form, E*. β-D-glucosidase was subject to competitive inhibition by glucose (K_G_^b^). A similar modeling approach done without consideration of potential effects of lignin was described previously (Scott *et al*., 2015).

The rate equations for the first (Equation 1) and second (Equation 2) stages of the model are shown below.

1 $$-\,\frac{{\rm{dS}}}{{\rm{dt}}}=\frac{{{\rm{k}}}_{{\rm{s}}}\cdot {{\rm{E}}}_{{\rm{a}}}\cdot {\rm{S}}}{{\rm{S}}+{{\rm{K}}}_{{\rm{s}}}(1+\frac{{\rm{G}}}{{{{\rm{K}}}_{{\rm{G}}}}^{{\rm{a}}}}+\frac{{\rm{G}}2}{{{\rm{K}}}_{{\rm{G}}2}}+\frac{L}{{K}_{L}})}=\frac{{\rm{dG}}2}{{\rm{dt}}}\times \frac{342\,{\rm{g}}/{\rm{mol}}}{324\,{\rm{g}}/{\rm{mol}}}$$

where,

S is the substrate cellulose (g/L);

t is time (h);

*k*_s_ is the cellulase catalytic rate constant (h^−1^);

E_a_ is the concentration of active cellulase (g/L);

K_s_ is the cellulase Michaelis-Menten constant (g/L);

G is glucose (g/L)

K_G_^a^ is the competitive glucose inhibition constant for cellulase (g/L)

G2 is cellobiose (g/L)

K_G2_ is the competitive cellobiose inhibition constant for cellulase (g/L)

L is lignin (g/L)

K_L_ is the competitive lignin inhibition constant for cellulase (g/L)

2 $$\frac{{\rm{dG}}}{{\rm{dt}}}=\frac{{k}_{{\rm{cat}}}\cdot \text{Bg}\,.{\rm{G2}}}{G2+{{\rm{K}}}_{{\rm{M}}}(1+\frac{{\rm{G}}}{{{{\rm{K}}}_{{\rm{G}}}}^{{\rm{b}}}})}\times \frac{360\,{\rm{g}}/{\rm{mol}}}{342\,{\rm{g}}/{\rm{mol}}}$$

where,

*k*_cat_ is the β-D-glucosidase catalytic rate constant (h^−1^);

Bg is the concentration of β-D-glucosidase (g/L);

K_M_ is the β-D-glucosidase Michaelis-Menten constant (g/L);

K_G_^b^ is the competitive glucose inhibition constant for β-D-glucosidase (g/L)

Inactivation of cellulases under the assay conditions described above was assumed to be first-order and is described in Equation 3. A cellulase performance half-life (t_1/2_) was calculated according to Equation 4. β-glucosidase activity was assumed to be stable.

3 $$\frac{{{\rm{dE}}}_{{\rm{a}}}}{{\rm{dt}}}=-{k}_{{\rm{i}}}\cdot {{\rm{E}}}_{{\rm{a}}}$$

where,

*k*_i_ is the cellulase inactivation rate constant (h^−1^)

4 $${{\rm{t}}}_{1/2}=\frac{0.693}{{k}_{{\rm{i}}}}$$

Potential challenges in applying Michealis-Menten kinetics to the hydrolysis of cellulose have been reported and some of these limtiations are addressed in the Discussion. Effects of glucose and cellobiose are modeled here as competitive inhibition although evidence supporting both competitive, non-competitive and uncompetivie cellulase inhibition has been reported (Reviewed in Andric *et al*., 2010). For comparative purposes, the data presented in Fig. 2 was also fit assuming that inhibition from glucose and cellobiose is non-competitive or uncompetivie. The effects of these changes on the trends in the parameter values shown in Table 3 were negligible (results not shown).

The differential equations were applied to each data set using a 4^th^ order Runge Kutta numerical integration using Microsoft Excel. Each experimental time course was divided into 1000 time steps that increased geometrically from t = 0 h. The concentrations of S, G2 and G were estimated four times for each time step using Equations 1 and 2 using the same estimated residual concentration of active cellulase enzyme calculated using Equation 3. The concentration of active cellulase was recalculated for each time step. The model was used to fit all doses of enzyme tested under a given set of experimental conditions by varying *k*_i_ and either *k*_s_ or K_L_. Optimal values of these parameters were determined simultaneously using the Excel Solver by minimization of least squares. All other parameters in the kinetic model were fixed to the values shown in Table 1. Two-dimensional 95% confidence intervals and standard deviations of the parameter values were calculated for each model fit. Student’s T-test was used to determine whether differences in parameter values were statistically significant. This information is included in Tables 2, 3, 5 and 6.

### Enzymatic digestions in the presence of small molecular weight lignins

Enzymatic digestions were carried out in 2.0 mL screw-cap vials at 1% solids loading at 75 and 45 °C while rotating end-over-end at 12 rpm for 96 h. Commercial enzyme preps were added at the level of 20 mg protein per gram of cellulose (corresponding to about 12 FPU/g of cellulose). No additional accessory enzymes were added except for β-D-glucosidase. The total volume of the saccharification slurries after adding enzyme and 50 mM citrate buffer was 2.0 mL. To determine the progress of cellulose conversion, 100 μL aliquots of the well-mixed slurries were taken at 8, 24, 48, 72, and 96 h. The samples were immediately diluted with 900 μL of DI water, and the enzymes were inactivated by heating at 95°C for 12 min. Samples were filtered through Pall Acrodisc nylon 0.2 µm syringe filters (Pall, Port Washington, NY) and refrigerated until HPLC analysis on an Agilent 1100 using a 300 mm × 7.8 mm BioRad Aminex HPX87 H ion exclusion column maintained at 55°C. The mobile phase was 0.01 N sulfuric acid at a flow rate of 0.6 mL/min. The sample injection volume was 20 µL and the run time 25 min. The cellulose conversion was calculated by adding the total glucose and cellobiose yields (both glucose and cellobiose were converted to glucan equivalent) for each hydrolysis time point.

### CelA and Cel7A incubated in the presence of lignin

Studies were done to determine the affinity of CelA has to lignin as well as Soluble low molecular weight lignin (SLMWL) using SDS-Page gel assays and to quantify the amount of protein loss and is compared with Cel7A. For the SDS-PAGE gel assays, CelA and Cel7A enzymes were incubated at 6 mg protein/g lignin (the amount of lignin that a protein to exposed to in a 20 mg protein/g biomass with 30% lignin content) and 1% solids loading at two temperatures, 30 °C and at their optimal temperature for activity (75 °C for CelA and 45 °C for Cel7A, respectively) to look at the temperature dependence of the absorption of these enzyme to lignin.

After incubation, the Cel7A at 45 °C and 30 °C was loaded onto a gel (Figure S6a) with the first lane being the molecular weight standards, lane 2, 6 and 10 are intentionally left blank, lane 3 is the control at 45 °C, lane 4 is the unbound fraction at 45 °C and lane 5 is the bound fraction 45 °C. Lane 7 through 9 are the same as lanes 3 through 5, but at 30 °C. Lanes 11 through 14 is the different Cel7A loading for the calibration curve. Figure S6b is CelA with the molecular weight standards in lane 1, lane 2 is the CelA control at 75 °C, lane 3 is the unbound fraction of CelA at 75 °C and lane 4 is the bound fraction of CelA at 75 °C to lignin. Lanes 6 through 8 are the same as lanes 2 through 4 but at 30 °C. Lanes 5 and 9 are intentionally left blank; and lanes 10 through 15 are the different CelA loadings for the calibration curve.

At first glance, it visually appears Cel7A (Figure S6b) has a lower affinity toward lignin at both temperatures in comparison to CelA (Figure S6a). Densitometry was performed on these gels to measure and quantify the amount of protein lost to lignin using Image J software^43, 16^.

A calibration curve between pixel density and total protein concentration was developed (Figure S5) of both CelA and Cel7A using the purified enzymes at varying concentration from 0.1 mg/ml to 1.2 mg/mL. From this standard curve, total protein amounts were calculated for the control and unbound fraction. Protein quantification was not performed on the bound fraction due to the increase background within the lane. Using Image J, the total protein numbers were calculated and shown in Fig. (3c). The protein loading for both CelA and Cel7A was targeted to 0.2 mg/mL and from the quantification, it can be seen that the average loading for CelA is 0.222 mg/mL.

### Biomass substrate microscopy

#### Stereomicroscopy

Samples of the pretreated, digested corn stover were examined directly without further processing. Images were captured on a Nikon SMZ1500 stereomicroscope and captured with a Nikon DS-Fi1 CCD camera operated by a Nikon Digital Sight system (Nikon Instruments, Melville, NY).

#### Fixation and Embedding

Digested, pretreated corn stover tissue was processed using microwave EM processing as reported previously^18^. Briefly, samples were fixed 2 × 6 min in 2.5% glutaraldehyde buffered in 0.1 M sodium cacodylate buffer (EMS, Hatfield, PS) under vacuum. The samples were dehydrated by treating with increasing concentrations of ethanol and heating in Pelco microwave oven for one min each dilution (i.e., 30%, 60%, 90%, and 3 × 100% ethanol). After dehydration, the samples were infiltrated with LR White resin (EMS, Hatfield, PA) by incubating at room temperature (RT) for several hours to overnight in increasing concentrations of resin (30%, 60%, 90%, 3 × 100% resin, diluted in ethanol). The samples were transferred to capsules and the resin polymerized by heating to 60 °C overnight in a vacuum oven.

#### Microtomy and Immuno-labeling

Resin-embedded samples were sectioned to ~60 nm with a Diatome diamond knife on a Leica EM UTC ultramicrotome (Leica, Wetzlar, Germany). Sections were collected on 0.5% Formvarcoated palladium/copper slot grids (SPI Supplies, West Chester, PA). Grids were placed on ~10 μp drops of 2.5% non-fat dry milk in 1X phosphate-buffered saline-0.1% Tween (PBST) for 30 min, then directly placed on ~10 lar, Germany). Section antibody probes diluted 1:50 in 1% milk PBST and incubated overnight at 4^o^C. Following a one min rinse with 1X PBST and 3 x one min rinses with nano-pure water, grids were placed on drops of buffered saline-0.1% Tween (PBST) for 30 min, the BioCell) diluted 1:500 in 1% milk PBST and incubated overnight at 4 diluted 1:500 in 1% milk PBST and incubated overnight at line-0.1% Tween (PBST) for nano-pure water.

#### Confocal Scanning Laser Microscopy (CSLM)

Semi-thin sectioned samples were positioned on glass microscope slides and stained with 0.1% acriflavine. Samples were excited at 488 nm and an emission range from 510–630 nm was captured. Images were captured using a 40× 1.4NA Plan Apo lenses on a Nikon C1 Plus microscope (Nikon, Tokyo, Japan), equipped with the Nikon C1 confocal system operated via Nikon’s EZ-C1 software.

#### Transmission Electron Microscopy (TEM)

Grids were post-stained for three minutes with 2% aqueous uranyl acetate and two minutes with 1% KMnO_4_ to selectively stain for lignins. Micrographs were captured with a four mega-pixel Gatan UltraScan 1000 camera (Gatan, Pleasanton, CA) on a FEI Tecnai G2 20 Twin 200 kV LaB6 TEM (FEI, Hilsboro, OR).

#### Image Analysis

Fiji (ImageJ) was used to rotate, crop, resize, and adjust contrast, brightness and white balance of images and to threshold images to aid in positively identifying gold nanoparticles for quantitation.

### Data and materials availability

All data needed to evaluate the conclusions in the paper are present in the paper and the Supplementary Materials or available upon request from the authors.

## Electronic supplementary material

Supplementary Materials
