# Supplementary material for: The Multi Domain Caldicellulosiruptor bescii CelA Cellulase Excels at the Hydrolysis of Crystalline Cellulose
Source: Sci Rep. 2017 Aug 29;7:9622. doi: 10.1038/s41598-017-08985-w (PMC5575103; doi:10.1038/s41598-017-08985-w)
Supplement: Supplementary file 1 — Supplementary Materials [file 41598_2017_8985_MOESM1_ESM.docx]

Supplementary Materials

The Multi Domain *Caldicellulosiruptor bescii* CelA Cellulase Excels

at the Hydrolysis of Crystalline Cellulose

**Authors**

Roman Brunecky^1^, Bryon S. Donohoe^1^, John M. Yarbrough^1^, Ashutosh Mittal^1^, Brian R. Scott^2^, Hanshu Ding^2^, Larry E. Taylor II^1^, Jordan F. Russell^3^, Daehwan Chung^1^, Jan Westpheling^3^, Sarah A. Teter^2^, Michael E. Himmel^1^, Yannick J. Bomble^[[1]](#footnote-1)^*

**Affiliations**

1. Biosciences Center, National Renewable Energy Laboratory, 15013 Denver West Parkway, Golden CO 80401, USA.
2. Novozymes, Inc., 1445 Drew Ave, Davis CA 95618, USA
3. Department of Genetics, University of Georgia, Athens GA 30602, USA.

**Fig. S1.** Arrow diagrams describing the two-stage Michaelis-Menten kinetic model with competitive lignin and product inhibition. A model describing the conversion of cellulose (S) to cellobiose (G2) catalyzed by active cellulase enzyme (E_a_) is shown in **(a)**. The cellulase in this model is subject to competitive inhibition by lignin (L), glucose (G) and cellobiose (G2). The cellulase is also subject to first-order inactivation to the inactive form (E*). The model process by which cellobiose is converted to glucose (G) by β-D-glucosidase (Bg) is shown in **(b)**. β-D-glucosidase as shown is subject to competitive inhibition by glucose.

**Fig. S2.** Model fits to the CelA and Cel7A progress curves on cotton linters. CelA (a) and Cel7A (b) were tested on cotton linters with varying crystallinity indices (CI). CelA was tested at 75°C and Cel7A was tested at 50°C. Model fits were done by varying *k*_s_ for each data set and selecting shared values of *k*_i_ for CelA and, separately, Cel7A. These parameter values are shown in Table 3. All other parameter values were fixed. Values plotted are fractional conversion of initial cellulose to glucose where 100% conversion=1.0.

**Fig. S3.** Model fits of differential biomass digestions by Cb broth and CTec2. Cb broth and CTec2 were tested on DACS (a), CFCS (b), APCS (c) and Avicel (d). Cb broth was tested at 75°C and CTec2 was tested at 50°C. The plots shown are the model fits to these progress curves generated by varying *k*_s_, *k*_i_ and/or K_L_ as described in the Results. The model parameters are shown in Table 2. Values plotted are fractional conversion of initial cellulose to glucose where 100% conversion=1.0

**Fig. S4.** Model fits to CelA progress curves on DACS in the presence of Tween 20, Tween 80 and BSA. CelA was incubated with DACS at 75°C in the presence of Tween 20 (a), Tween 80 (b) and BSA (c). Model fits were generated by varying K_L_ and *k*_i_ and these parameter values are shown in Table 5. Values plotted are fractional conversion of initial cellulose to glucose where 100% conversion=1.0


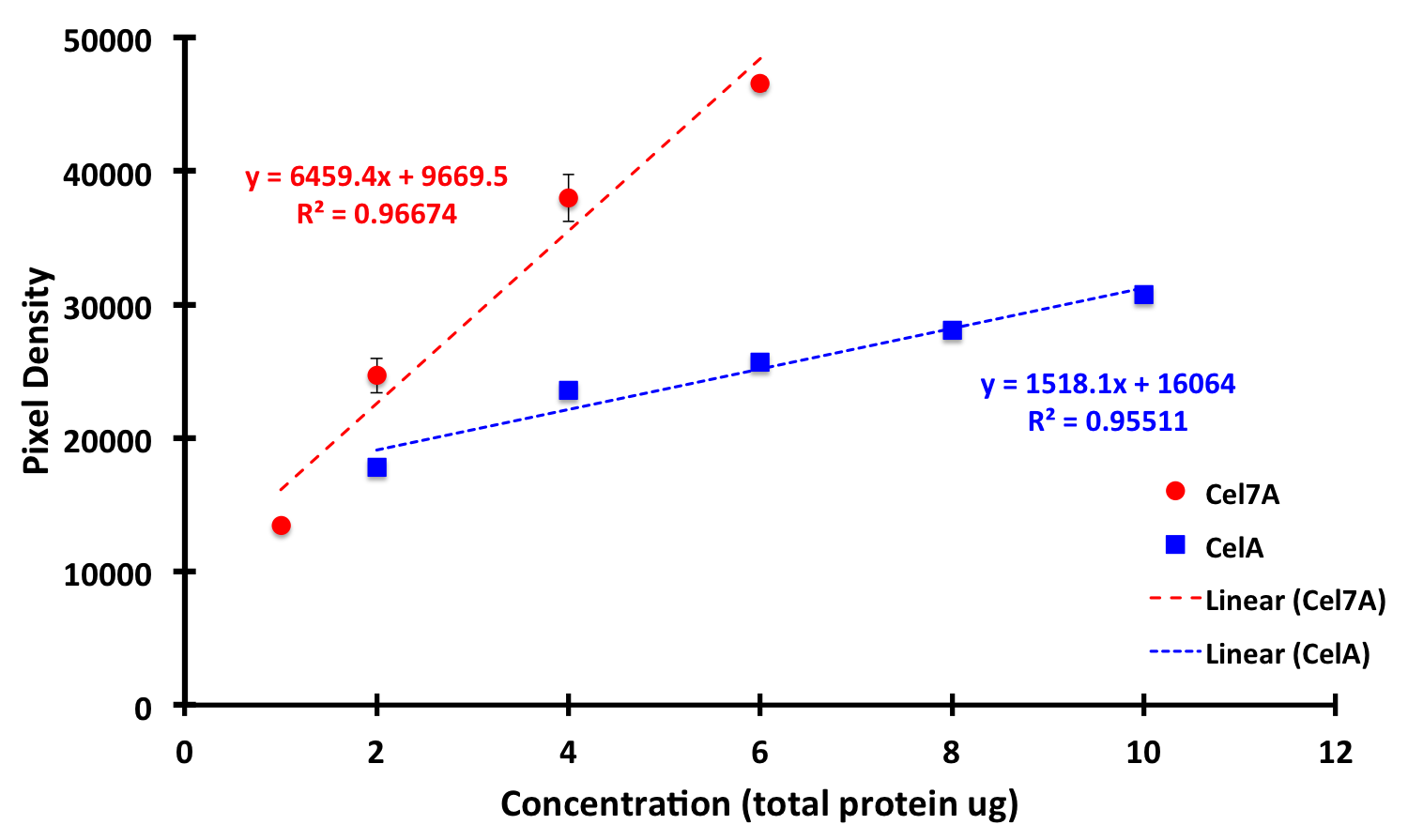


**Fig. S5**. Calibration curves for CelA (blue) and Cel7A (red) for quantification of protein concentration. These curves were developed using gel analysis software in Image J and used to measure the amount of protein in a given band.

**
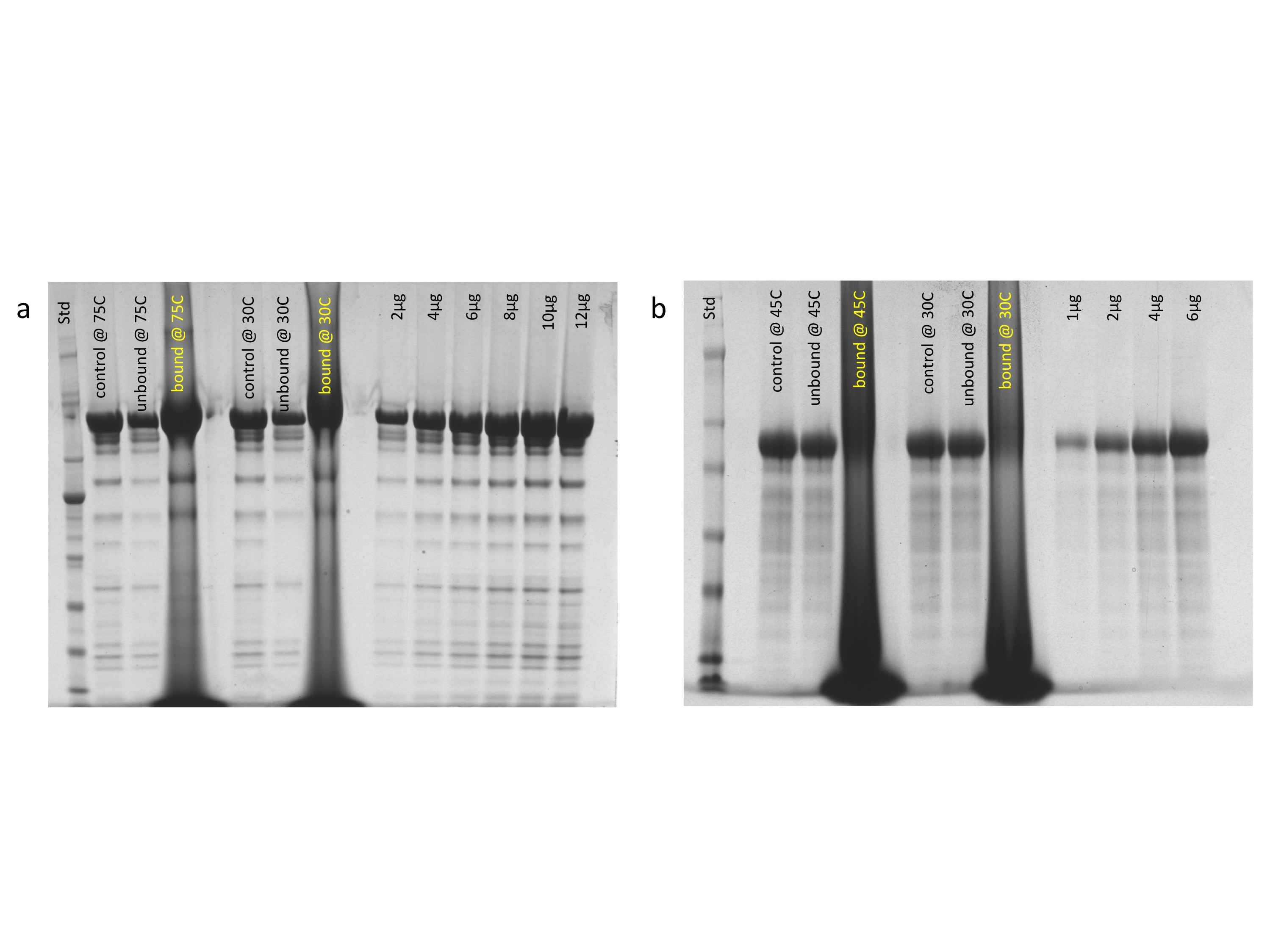
**

**Fig. S6**. SDS-Page Gel of CelA (a) and Cel7A (b) for density measurements with the following lane assignments, lane 1: STD, lane 2: protein control at running temperature, lane 3: unbound protein fraction at running temperature, Lane 4: bound protein fraction with lignin, Lane 5: protein control at 30°C, lane 6: unbound protein fraction at 30°C, Lane 7: bound protein fraction at 30°C and lanes 8-15 are the protein concentration ladder used to calculate protein concentration.

**Table S1.** Compositions of Avicel and DA, AP and CF pretreated corn stover.

|  | Glucan  (%) | Xylan  (%) | Lignin  (%) | Galactan  (%) | Arabinan  (%) | Fructan  (%) | Acetate  (%) |
| --- | --- | --- | --- | --- | --- | --- | --- |
| Dilute Acid | 63.9 | 5.0 | 23.9 | 0.6 | 0.8 | 0 | 0.2 |
| Alkaline Peroxide | 55.6 | 33.4 | 3.9 | 1.2 | 2.9 | 0.4 | 0.1 |
| Clean Fractionated | 88.4 | 2.3 | 3.3 | 0.7 | 0 | 0.6 | 0.1 |
| Avicel | 95 | 1 | 0 | nd | nd | nd | nd |

1. [↑](#footnote-ref-1)
